# Predicting the success of an invader: Niche shift versus niche conservatism

**DOI:** 10.1002/ece3.5734

**Published:** 2019-10-25

**Authors:** Stéphanie Sherpa, Maya Guéguen, Julien Renaud, Michael G. B. Blum, Thierry Gaude, Frédéric Laporte, Mustafa Akiner, Bulent Alten, Carles Aranda, Hélène Barre‐Cardi, Romeo Bellini, Mikel Bengoa Paulis, Xiao‐Guang Chen, Roger Eritja, Eleonora Flacio, Cipriano Foxi, Intan H. Ishak, Katja Kalan, Shinji Kasai, Fabrizio Montarsi, Igor Pajović, Dušan Petrić, Rosa Termine, Nataša Turić, Gonzalo M. Vazquez‐Prokopec, Enkelejda Velo, Goran Vignjević, Xiaohong Zhou, Laurence Després

**Affiliations:** ^1^ Laboratoire d'Ecologie Alpine (LECA) CNRS Université Grenoble Alpes Grenoble France; ^2^ Laboratoire Techniques de l'Ingénierie Médicale et de la Complexité (TIMC‐IMAG) CNRS Université Grenoble Alpes Grenoble France; ^3^ Department of Biology Faculty of Arts and Sciences Recep Tayyip Erdogan University Fener Turkey; ^4^ Vector Ecology Research Group (VERG) Ecological Sciences Research Laboratories Department of Biology Faculty of Science Hacettepe University Ankara Turkey; ^5^ Centre de Recerca en Sanitat Animal (CReSA IRTA) Barcelona Spain; ^6^ Servei de Control de Mosquits Consell Comarcal del Baix Llobregat Barcelona Spain; ^7^ Observatoire Conservatoire des Insectes de Corse Office de l'Environnement de la Corse Corti France; ^8^ Department of Medical and Veterinary Entomology Centro Agricoltura Ambiente “G.Nicoli” Crevalcore Italy; ^9^ Consultoria Moscard Tigre Palma de Mallorca Spain; ^10^ Department of Pathogen Biology School of Public Health Southern Medical University Guang Zhou China; ^11^ Laboratorio Microbiologia Applicata Dipartimento Ambiente Costruzioni e Design Scuola Universitaria Professionale della Svizzera Italiana Porza Switzerland; ^12^ Istituto Zooprofilattico Sperimentale della Sardegna “G. Pegreffi” Sassari Italy; ^13^ School of Biological Sciences Universiti Sains Malaysia Penang Malaysia; ^14^ Department of Biodiversity Faculty of Mathematics, Natural Sciences and Information Technologies University of Primorska Koper Slovenia; ^15^ Department of Medical Entomology National Institute of Infectious Diseases Tokyo Japan; ^16^ Laboratory of Parasitology Istituto Zooprofilattico Sperimentale delle Venezie Padova Italy; ^17^ University of Montenegro Biotechnical Faculty Podgorica Montenegro; ^18^ Laboratory for Medical and Veterinary Entomology Faculty of Agriculture University of Novi Sad Novi Sad Serbia; ^19^ Laboratorio di Ingegneria Sanitaria Ambientale Università “Kore” di Enna Enna Italy; ^20^ Department of Biology Josip Juraj Strossmayer University Osijek Croatia; ^21^ Department of Environmental Sciences Emory University Atlanta GA USA; ^22^ Department of Epidemiology and Control of Infectious Diseases Institute of Public Health Tirana Albania

**Keywords:** *Aedes albopictus*, ecological niche modeling, generalized dissimilarity modeling, genotype–environment association, geometric morphometrics, niche conservatism, RAD sequencing, rapid adaptation

## Abstract

Invasive species can encounter environments different from their source populations, which may trigger rapid adaptive changes after introduction (niche shift hypothesis). To test this hypothesis, we investigated whether postintroduction evolution is correlated with contrasting environmental conditions between the European invasive and source ranges in the Asian tiger mosquito *Aedes albopictus*. The comparison of environmental niches occupied in European and source population ranges revealed more than 96% overlap between invasive and source niches, supporting niche conservatism. However, we found evidence for postintroduction genetic evolution by reanalyzing a published ddRADseq genomic dataset from 90 European invasive populations using genotype–environment association (GEA) methods and generalized dissimilarity modeling (GDM). Three loci, among which a putative heat‐shock protein, exhibited significant allelic turnover along the gradient of winter precipitation that could be associated with ongoing range expansion. Wing morphometric traits weakly correlated with environmental gradients within Europe, but wing size differed between invasive and source populations located in different climatic areas. Niche similarities between source and invasive ranges might have facilitated the establishment of populations. Nonetheless, we found evidence for environmental‐induced adaptive changes after introduction. The ability to rapidly evolve observed in invasive populations (genetic shift) together with a large proportion of unfilled potential suitable areas (80%) pave the way to further spread of *Ae. albopictus* in Europe.

## Graphical abstract

Invasive species can encounter environments different from their source populations, which may trigger rapid adaptive changes after introduction. Combining niche distribution modeling, genotype–environment associations and generalized dissimilarity modeling, we found evidence for environmental‐induced adaptive changes in the Asian tiger mosquito *Aedes albopictus* after its introduction in Europe. The ability to rapidly evolve observed in invasive populations together with a large proportion of unfilled potential suitable areas pave the way to further spread of *Ae. albopictus* in Europe.

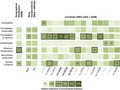

## 1 INTRODUCTION

Human movements have dramatically increased in the past decades, promoting the intentional or accidental introduction of species into new regions often far removed from their natural ranges (Banks, Paini, Bayliss, & Hodda, 2015; Hulme, 2009). Predicting the potential risks of establishment and spread of non‐native species has thus become a central question in invasion biology (Jiménez‐Valverde et al., 2011; Thuiller et al., 2005). The commonly used approach is to predict the potential range of an invasive species using environmental characteristics of known geographic occurrences in its native range (Guisan & Thuiller, 2005; Soberon & Peterson, 2005). A fundamental assumption in these predictions is that invasive species retain their ancestral ecological niche (i.e., niche conservatism; Wiens & Graham, 2005) and it makes it difficult to predict the introduced range from the species' native range if the non‐native and native niches differ (i.e., niche shift; Broenniman et al., 2007).

It has become increasingly important to evaluate whether the ecological characteristics of species are maintained or change rapidly when they establish outside their initial range (Guisan, Petitpierre, Broennimann, Daehler, & Kueffer, 2014; Pearman, Guisan, Broennimann, & Randin, 2008). A growing number of studies report such niche shift during the invasion process (Atwater, Ervine, & Barney, 2018; Broenniman et al., 2007; Guisan et al., 2014; Lancaster, Dudaniec, Hansson, & Svensson, 2015; Petitpierre et al., 2012). Global occurrences are classically used as a background for predicting the potential distribution of species. However, comparing the ecological characteristics of the invaded and the full species' native ranges may be misleading if the introduced populations do not directly originate from the native but another invasive range (i.e., bridgehead effect; Lombaert et al., 2010). Elucidating the routes of introduction is a prerequisite to adequately address the question of niche shift versus niche conservatism during the invasion process. Population genomics and modern analytical tools, such as approximate Bayesian computation, allow to combine historical, biological, and genetic information, to test for complex scenarios including demographic stochasticity (i.e., bottleneck) and multiple introductions (i.e., genetic admixture), and provide decision statistics to choose the most likely scenario (Estoup & Guillemaud, 2010).

The study of the geographical distribution of invasive species can also provide valuable information about their invasiveness. For instance, niche similarity between non‐native and native ranges may favor the rapid establishment of introduced populations. However, niche expansion requires local adaptation that ultimately determines the capacity of populations to persist (Richardson & Pyšek, 2008; Sax et al., 2007), raising further questions about the evolutionary mechanisms at play during the invasion process. How fast do populations evolve in response to new selective pressures? To what extent does the demographic history (e.g., genetic admixture, founder events) account for local adaptation? Is there a causal link between niche conservatism and range expansion? Ecological genomics approaches now allow characterizing the role of environmental variables in shaping local adaptation (Ahrens et al., 2018; Hoban et al., 2016; Rellstab, Gugerli, Eckert, Hancock, & Holderegger, 2015). For invasive species undergoing range expansion, genes essential for local adaptation are expected to present shifts in allele frequencies along environmental gradients (Dudaniec, Yong, Lancaster, Svensson, & Hansson, 2018; Fitzpatrick & Keller, 2015). Other traits such as morphological, physiological, or life‐history traits that often show heritable variation may also evolve rapidly in response to new selective pressures (Lynch & Walsh, 1998; Nosil, 2012; Thompson, 1998).

Among recent biological invasions, the Asian tiger mosquito, *Aedes* (*Stegomyia*) *albopictus* (Skuse 1894), has been the focus of a large number of species distribution modeling studies at various spatial scales (Caminade et al., 2012; Dickens, Sun, Jit, Cook, & Carrasco, 2018; Ducheyne et al., 2018; ECDC, 2009, 2012; Fischer, Thomas, Niemitz, Reineking, & Beierkuhnlein, 2011; Kraemer et al., 2015; Medlock, Avenell, Barrass, & Leach, 2006; Roiz, Neteler, Castellani, Arnoldi, & Rizzoli, 2011). These studies depicted a consensus of the geographical determinants of *Ae. albopictus* global distribution range, but they primarily aimed at evaluating the potential contemporary and future distributions. Studies evaluating the niche conservatism hypothesis revealed that invaded niches differ from those of native populations (Cunze, Kochmann, Koch, & Klimpel, 2018; Hill, Gallardo, & Terblanche, 2017; Medley, 2010). These differences were either explained by niche expansion supporting niche shift (Hill et al., 2017) or by niche unfilling supporting niche conservatism (Cunze et al., 2018). These studies compared invaded range niches to those of the entire Asian native range (Cunze et al., 2018; Medley, 2010), but assessing the adaptive potential of introduced populations requires having a good knowledge of their precise source. For instance, the reconstruction of *Ae. albopictus* invasion routes has revealed that the sources can be previously invaded areas (Sherpa, Blum, Capblancq, et al., 2019).

In the present study, we address the question of niche shift versus niche conservatism during the invasive range expansion of *Ae. albopictus* in Europe, by comparing the ecological characteristics of European invasive populations to the characteristics of their North American and Chinese source populations (Sherpa, Blum, Capblancq, et al., 2019). Because North American populations originated in Japan, where preexisting cold adaptation probably favored invasion in temperate regions (Sherpa, Blum, Capblancq, et al., 2019; Sherpa, Blum, & Després, 2019), we also included Japan in niche comparisons. The examination of niche shifts during the invasion process of *Ae. albopictus* has never been combined so far to the analysis of traits that may affect local adaptation. We thus tested whether substantial differences in environmental niches occupied by European invasive populations may have promoted rapid adaptive changes after introduction. We used published genomic data from double‐digest restriction‐associated DNA sequencing (ddRADseq) from 90 populations distributed throughout the European invasive range and measured wing geometric morphometrics for a subset of these populations. To evaluate the ability of European populations to evolve in response to new selective pressures, we tested the effect of six environmental factors on genetic composition and morphometric variation using correlative approaches controlling for populations demographic history. Traits potentially under current selection within Europe were then compared to their source populations.

## 2 MATERIAL AND METHODS

### 2.1 Study area

**Figure 1:**
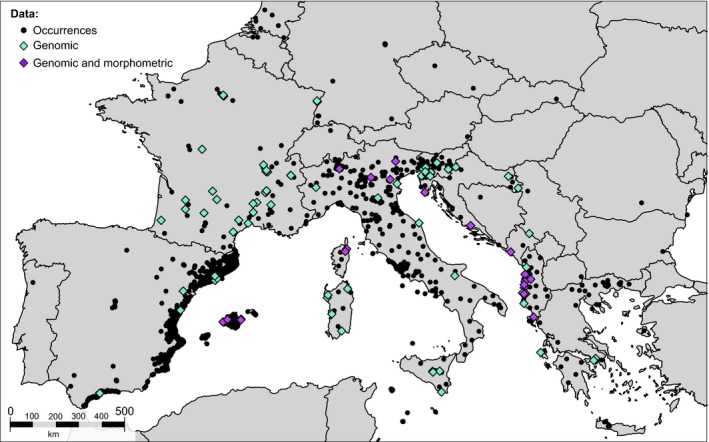
Data collection. Distribution data of *Aedes albopictus* comprise 4,649 occurrences from freely available online databases and literature review of previous distribution studies or sample material (Table S2). ddRADseq genomic data comprise 90 localities (*N* = 551; Table S1) from previously published data (Table S3; Sherpa, Blum, Capblancq, et al., 2019). Morphometric data comprises 19 populations (*N* = 238) generated for the purpose of this study

The study area encompasses 90 invasive populations distributed across the current European invasive range of *Ae. albopictus*, which have been analyzed in a previous study (Sherpa, Blum, Capblancq, et al., 2019). The samples include populations from Albania (11), Croatia (3), France (30, including 2 from Corsica), Greece (3, including 1 from Kefalonia), Italy (17, including 5 from Sardinia and 4 from Sicily), Montenegro (1), Serbia (2), Slovenia (10), Spain (11, including 4 from Majorca), and Switzerland (2) (Figure 1, Table S1). Based on the previous reconstruction of colonization routes, we included data from the two European source populations (China and United States) and Japan (ancestral origin of United States; Figure S1; Sherpa, Blum, Capblancq, et al., 2019).

### 2.2 Data collection

#### 2.2.1 Occurrence data

We collected occurrences (presence only records) of *Ae. albopictus* available from the Global Biodiversity Information Facility (https://www.gbif.org/) and the citizen science project (https://www.inaturalist.org/), and literature review of previous distribution studies or sample material (Table S2). *Aedes albopictus* has been introduced in North Italy from the United States in 1990 (Sabatini, Raineri, Trovato, & Coluzzi, 1990; Sherpa, Blum, Capblancq, et al., 2019) so we considered only the occurrences recorded before 1990 for the US source in order to compare the ecological characteristics of US invasive populations before their introduction into Europe. We retained uniquely georeferenced occurrences, resulting in 4,649 occurrences in Europe, 265 in China, 81 in Japan, and 275 in the United States (Figure 1, Figure S1).

#### 2.2.2 Environmental data

Climatic conditions are determinant for *Ae. albopictus* adult activity, egg development, and egg overwintering survival. Environmental predictors for its establishment classically include annual mean temperature (suitable above 11°C), annual precipitations (suitable above 500 mm), and minimum temperature of the coldest month (suitable above 0°C; Fischer, Thomas, Neteler, Tjaden, & Beierkuhnlein, 2014). We collected climatic data from the CHELSA database v1.2 (http://chelsa-climate.org) at a resolution of 30 arc seconds, with monthly mean temperature and precipitation averaged over the period 1979–2013 (Karger et al., 2017). We included the 19 yearly bioclimatic variables and mean monthly precipitation, and mean, minimum, and maximum temperatures. We also retrieved the Global‐Aridity Index, related to evapotranspiration processes and rainfall deficit, from the CGIAR‐CSI Database (http://www.cgiar-csi.org) at a resolution of 30 arc seconds for the period 1970–2000 (Zomer, Trabucco, Bossio, & Verchot, 2008). We considered two indices from the NASA Socioeconomic Data and Applications Center (SEDAC; https://sedac.ciesin.columbia.edu): the net primary productivity (NPP; Imhoff et al., 2004) and the 2009 human footprint (HF) because *Ae. albopictus* is an anthropophilic species. HF is a cumulative index indicating human pressure on the environment measured using eight variables (built‐up environments, population density, electric power infrastructure, croplands, pasture lands, roads, railways, and navigable waterways) at a resolution of ~1 km (Venter et al., 2018). We retained noncolinear variables using the occurrences in the four geographical regions (correlation coefficients < 0.50), resulting in six variables to represent environmental variation among ranges: PRJ (precipitation in January), PRS (precipitation seasonality), MTP (minimum temperature of the coldest month), ISO (isothermality), NPP (net primary production), and HF (human footprint; Figure S2).

#### 2.2.3 Genetic data acquisition

We reanalyzed previously published genomic data obtained from double‐digest restriction‐associated DNA sequencing (ddRADseq; Sherpa, Blum, Capblancq, et al., 2019; Sherpa, Rioux, Pougnet‐Lagarde, & Després, 2018). Sequences of 110 bp for 90 European populations (*N* = 551) were mapped to the *Ae. albopictus* reference genome (Chen et al., 2015) using BWA‐MEM v0.7.5 (Li et al., 2009). We retained uniquely aligned reads with MapQ ≥ 30 using SAMTOOLS v1.7 (Li & Durbin, 2009), and with a minimum read depth of 5 reads/individual on average using STACKS v.2.0 (Catchen, Hohenlohe, Bassham, Amores, & Cresko, 2013). We included all polymorphic positions, with a maximum of 30% missing data and a minor allele count of 40, resulting in a dataset of 6,506 SNPs with 18.5% missing data.

#### 2.2.4 Mosquito wings

The characterization of wing morphometric variation was performed on laboratory‐raised individuals to report morphometric differences that reflect genetic differences among populations and measured the first generation so that the phenotypic characteristics of populations are not altered. Eggs were reared in standard laboratory conditions (27°C, 70% relative humidity, and day length cycles of 14:10‐hr light:dark), with <1 larva per milliliter. Morphometric data were collected for 410 individuals from 25 invasive populations from Europe and 1 invasive population from United States (Table S1). We also included morphometric data from 153 individuals from 4 native populations (China and Japan) reared in the same laboratory conditions, analyzed in a previous study (Sherpa, Blum, & Després, 2019).

### 2.3 Environmental niche comparison

#### 2.3.1 Ordination method

Environmental niche variation of the six variables retained was evaluated using principal component analysis (PCA) implemented in the *ade4* package v1.7‐13 (Chessel, Dufour, & Thioulouse, 2004) in R v3.3.3 (R Core Team, 2017) for all analyses. We assessed the variation between the European invasive range niche and one of each source population by comparing the coordinates of occurrences in a two‐dimensional environmental space. We examined the coordinates of primarily introduced populations in Europe (Albania, North Italy, and Central Italy; Sherpa, Blum, Capblancq, et al., 2019) compared to populations outside Europe (China, Japan, United States), and the coordinates of primary versus subsequent introductions in Europe. We then calculated three niche metrics using occurrence densities along two gridded environmental gradients (i.e., the two first axes of PCA; Broennimann et al., 2012; Petitpierre et al., 2012) in the *ecospat* package v3.0 in R (Broennimann, Cola, & Guisan, 2018; Di Cola et al., 2017). We evaluated the niche overlap using Schoener's *D* (Schoener, 1968) and tested whether niches are more similar (similarity test) or different (equivalency test) than random expectation (Broennimann et al., 2012; Glennon, Ritchie, & Segraves, 2014; Warren, Glor, & Turelli, 2008). For similarity tests, we fixed the source niche as the reference and shifted only the European invasive niche. The significance of similarity and equivalency tests was assessed by 1,000 permutations.

#### 2.3.2 Niche‐based distribution modeling

We performed four niche distribution models in relation to the six environmental variables retained for each studied area (Japan, China, United States, Europe). To reduce spatial autocorrelation, one presence was randomly selected when several points fell within the same raster cell. For each region, five datasets of 5,000 pseudo‐absences were selected using a surface range envelope model. An ensemble of projections of species distributions models (SDM) from five statistical models was obtained, including generalized linear models (GLM), generalized additive models (GAM), boosted regression trees (BRT), multiple adaptive regression splines (MARS), and Random Forest (RF; Araújo & New, 2007; Marmion, Parviainen, Luoto, Heikkinen, & Thuiller, 2009; Thuiller, 2004). Models were calibrated for the baseline period using 70% of observations randomly sampled from the initial data and evaluated against the remaining 30% data using the true skill statistic (TSS, Allouche, Tsoar, & Kadmon, 2006) and the area under the curve (ROC, Swets, 1988). This analysis was repeated three times, thus providing threefold internal cross‐validation of the models. Models and the ensemble forecasting procedure were performed using the BIOMOD package (Thuiller, 2003; Thuiller, Lafourcade, Engler, & Araújo, 2009) implemented in the *biomod2* package v3.3‐7.1 in R (Thuiller, Georges, Engler, & Beriner, 2019).

The relative importance of each environmental variable was assessed by calculating the Pearson's correlation between the standard predictions (i.e., fitted values) and the predictions after randomly permuting the values of the variable. All calibrated models were then projected under current conditions over Europe to calculate the percentage of agreement between the potential European invasive range calibrated in Europe and the potential European invasive range calibrated in each source population. We evaluated niche stability (i.e., European range that overlaps between the two models), niche expansion (i.e., European range that is not predicted by the model calibrated in the source), and niche unfilling (i.e., European range that is not predicted by the model calibrated in Europe).

### 2.4 Genomic signature of selection

#### 2.4.1 SNP–environment association

Genotype–environment associations (GEA) were performed for testing association between each of the 6,506 SNPs and the six environmental variables. We used two GEA: the univariate latent factor mixed model (LFMM; Frichot, Schoville, Bouchard, & François, 2013) implemented in the *LEA* package v1.4.0 in R (Frichot & François, 2015), and the multivariate approach based on redundancy analysis (RDA; Capblancq, Luu, Blum, & Bazin, 2018; Forester, Lasky, Wagner, & Urban, 2018) using *rda* in the *vegan* package v2.4‐5 in R (Legendre & Legendre, 2012; Oksanen et al., 2017). The univariate GEA method LFMM tests for association between each SNP allele frequency and a single environmental predictor (Frichot et al., 2013). One‐factor LFMM models were run with five repetitions and 10,000 iterations with a burn‐in period of 2,000 iterations. The multivariate RDA decomposes genetic variance on a set of orthogonal axes in relation to several environmental predictors to find SNPs that covary with multivariate environmental patterns (Capblancq et al., 2018; Forester et al., 2018). SNPs are modeled as a function of predictor variables, producing as many constrained axes as environmental predictors. We aimed at finding overlapping SNPs between the two GEA methods, so we built a RDA model with all six environmental variables as predictors to identify SNPs correlated with each of the six constrained axes.

We accounted for the shared demographic histories of populations when modeling associations between SNPs and environmental predictors (i.e., at least three independent introduction events in Europe; Sherpa, Blum, Capblancq, et al., 2019). LFMM directly controls for population genetic structure using latent factors (Frichot et al., 2013) and were run with three latent factors. RDA does not directly model this confounding effect, but partial RDA allows integrating supplementary predicting variables as conditional, thus summarizing the component of genetic variation that is only explained by environmental variables. We used the ancestry coefficients for *K* = 3 genetic groups estimated in Sherpa, Blum, Capblancq, et al. (2019).

For LFMM, the *Z*‐scores of one‐factor models were combined and used to compute *p*‐values that were adjusted using a genomic inflation factor (GIF; Frichot & François, 2015). As recommended, we assessed the closeness of GIF (calculated from the *Z*‐scores derived) to the value of 1.0, which ranged from 1.12 to 1.73 across the six environmental variables. For the RDA, we used the loadings of each SNP in the ordination space (i.e., SNP scores) on constrained axes as a statistic for testing the significance of the correlation between SNPs and environmental data (Capblancq et al., 2018; Forester et al., 2018). The false discovery rate (FDR) control algorithm was applied to reduce the proportion of false positives detected by each GEA method (Storey & Tibshirani, 2003) using the *qvalue* package v2.4.2 in R (Storey, Bass, Dabney, & Robinson, 2015). We detected a set of loci (220 base sequences) among which several SNPs were discovered by at least one GEA method with a *Q*‐value threshold of 0.05 but retained only overlapping SNPs as “outlier SNPs.”

#### 2.4.2 Generalized dissimilarity modeling of candidate SNPs

We tested whether outlier SNPs show spatially explicit shifts in allele frequencies using generalized dissimilarity modeling (GDM; Fitzpatrick & Keller, 2015), implemented in the *gdm* package v1.3.11 in R (Ferrier, Manion, Elith, & Richardson, 2007; Manion et al., 2017). This method models the response of SNPs along environmental gradients by estimating the magnitude of change in allele frequency (i.e., allelic turnover; Fitzpatrick & Keller, 2015). Outlier SNPs located among different loci were modeled independently but together when located on the same locus. GDM uses population genetic distance matrices (pairwise *F*_ST_ for each SNP locus among populations). We subsampled our genetic dataset to only include populations with a minimum sample size of *N* ≥ 5 to obtain accurate allele frequencies. For each candidate SNP locus, 47 to 59 sample sites were analyzed. Pairwise *F*_ST_ among populations (Weir & Cockerham, 1984) for each SNP locus were calculated using *hierfstat* package v0.04‐22 in R (Goudet, 2005) and were rescaled between 0 and 1.

We applied three criteria to discover “candidate loci” associated with one environmental predictor among GEA outliers. First, we tested whether the allelic turnover at a given locus differs from random expectations. We randomly sampled 200 SNPs among the 6,506 available (i.e., reference group; Fitzpatrick & Keller, 2015) and evaluated whether the percentage of GDM deviance (%GDM) was higher than for the reference group. Second, we tested whether allelic variation at this locus is better explained by environment than isolation by distance. We included Euclidean geographic distance between populations in the GDM (Fitzpatrick & Keller, 2015) and evaluated whether the allelic turnover induced by the environment was higher than by geography. Loci that do not match this criterion were considered as false positives. Third, we evaluated whether one relevant environmental predictor influences the allelic turnover at this locus relative to other environmental predictors, with %GDM explained by that predictor ≥40%.

#### 2.4.3 Adaptive genetic variation maps

We screened genes with candidate loci using the VectorBase biomart online tool (https://biomart.vectorbase.org/biomart/martview/) and annotations AaloF1.2 of *Ae. albopictus* reference genome. When genes were not annotated, we evaluated orthologous genes in the VectorBase database. Candidate loci associated with one environmental predictor (GDM) and located in genes were further considered as putative “adaptive loci,” and their function was assessed using Gene Ontology annotations of the Universal Protein Knowledgebase (UniProt, http://www.uniprot.org).

The observed variation in allele frequencies restricted to adaptive loci was mapped against its expected adaptive genetic variation in Europe using the *gdm* v1.3.11 and *raster* v2.4.8 packages in R (Ferrier et al., 2007; Hijmans, 2018). In order to determine whether adaptive genetic polymorphisms within Europe were already present before introduction, we also used ddRADseq genomic data of source populations (China, United States, and Japan; Sherpa, Blum, Capblancq, et al., 2019). We exported the genotypes and computed allele frequencies of putative adaptive loci. The presence of the adaptive alleles in source populations but with different allele frequency relative to European invasive populations was indicative of postintroduction changes in response to new selective pressure (a relevant predictor of GDM).

### 2.5 Wing morphometric variation

#### 2.5.1 Landmark‐based geometric morphometrics

Wing morphometrics were performed using landmark‐based (LM) geometric morphometrics. Twenty LM located at vein intersections and termini of left wings (Figure S3; Sherpa, Blum, & Després, 2019) were digitalized using tpsUtil v1.76 (Rohlf, 2006) and tpsDig2 v2.31 (Rohlf, 2008). Variation due to scale, orientation, and position was removed by applying a Procrustes superimposition using IMP CoordGen8 (Sheets, 2008).

#### 2.5.2 Morphometric differentiation

Wing shape (LM Procrustes coordinates) variation among populations was first evaluated using the principal component analysis (PCA) in IMP CoordGen8 (Sheets, 2008). The level of morphometric differentiation was tested using analysis of variance models in the *car* package v3.0‐2 in R (Fox & Weisberg, 2011). Wing size (log‐transformed centroid size, CS) and shape (LM Procrustes coordinates) differentiation were tested using univariate analyses of variance (type II ANOVA) and multivariate analyses of variance (type II MANOVA), respectively. MANOVA and ANOVA were performed separately for males and females. In order to compare source and invasive populations, we first tested differences among main geographical regions (China, Japan, United States, Albania, North Italy, Corsica, Croatia, Majorca, Montenegro, Switzerland). Pairwise comparisons among populations were performed using a subset of the morphometric dataset because the sample size was small for some populations (Table S1). For each sex, we only tested differences for populations with at least five individuals.

#### 2.5.3 Environmental correlations

The effect of each environmental variable on wing size and shape was assessed using RDA, as implemented in the *vegan* package v2.4.5 in R (Oksanen et al., 2017). We first built a global model with morphometric data as response variables and the six environmental variables as explanatory variables to evaluate the proportion of morphometric variation that is constrained by the environment. Then, we ran one‐factor RDA models with each environmental variable and used the proportion of variance explained by each environmental variable to represent the relative importance of each environmental variable in morphometric variation. The significance of each fitted one‐factor model was assessed using ANOVA. As for RDA‐based GEA, all the models were fitted using a supplementary explanatory variable as conditional, removing the confounding effect of population genetic structure. Although morphometric data were obtained for the same sampled populations, individual data did not match between morphometric and genotypic datasets. Thus, we used the ancestry coefficients for *K* = 3 genetic groups estimated in Sherpa, Blum, Capblancq, et al. (2019) averaged per population.

## 3 RESULTS

### 3.1 Niche spaces of reduced dimensionality

**Figure 2:**
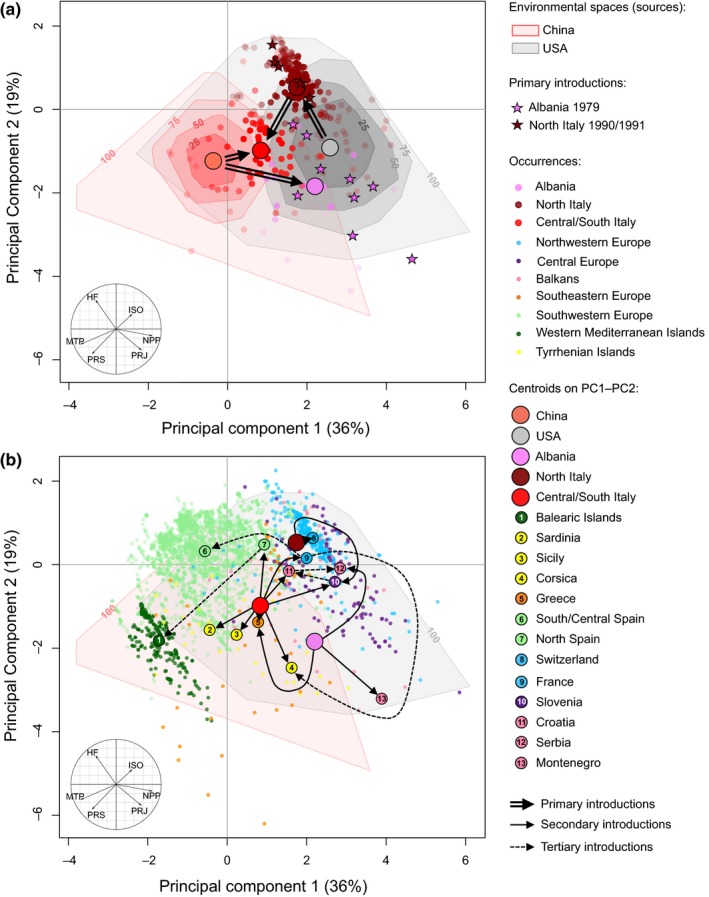
Environmental space comparisons. Comparisons are between environmental spaces of source and invaded ranges in (a), and primary introductions and subsequent introductions in Europe in (b). The convex hulls indicate the prevalence (25%, 50%, 75%, and 100% of sites included) of the environmental conditions in the source population ranges (United States, China). The stars show the position of the first introduction records in Europe. Occurrences are indicated with small dots and centroids with big dots (only for countries with reconstructed colonization routes; Sherpa, Blum, Capblancq, et al., 2019). Black arrows linking centroids represent the origin of source populations. The correlation circle indicates the importance of environmental variables on the two first axes of the PCA (55% of the total variance): PRJ (precipitation in January), PRS (precipitation seasonality), MTP (minimum temperature of the coldest month), ISO (isothermality), NPP (net primary production), and HF (human footprint)

The PCA of 5,270‐pooled occurrences (Europe, Japan, United States, China) for six environmental variables (PRS: precipitation seasonality, PRJ: precipitation in January, ISO: isothermality, MTP: minimum temperature of the coldest month, NPP: net primary production, HF: human footprint) revealed two significant axes of environmental variation (Figure 2). The first axis (PC1, 36% of total variance) differentiated the environmental spaces of source populations, with colder winters in China than in the United States (Figure 2a; see Figure S4 for Japan). The second axis (PC2, 19% of total variance) was mostly associated with HF. Niche centroids differed between source and European introduced ranges (Figure 2a), as well as between secondary and primary introductions in Europe (Figure 2b). Primary introduced populations in Albania show a niche shift that occurs along PC1 (Chinese source), indicating winter climate (MTP and PRJ) as the best predictors of niche differentiation (Figure 2a). Populations in North Italy occupy a similar climatic niche to their North American source (PC2) but an upward shift of the niche centroid can be observed, which is associated with a higher influence of human activities. The third primary introduced area (Central Italy) revealed a shift of the niche centroid that seems to result from a combination of the climate niches of the two source populations (North Italy and China). Several secondary introductions also show a shift of the niche centroid but the environmental space occupied by *Ae. albopictus* in Europe largely overlaps the Chinese and/or North American environmental spaces (Figure 2b).

The coordinates of occurrences on the two PCA axes were used as a representation of the realized niche space for each region to investigate niche conservatism between European invasive populations and source populations (United States, China, Japan). The European invasive range niche shows overlap with the source populations range niches with Schoener's *D* of 0.292 (Europe–United States), 0.334 (Europe–China) and 0.362 (Europe–Japan; *D* = 0: no overlap, *D* = 1: complete overlap). Despite niche overlap between environmental spaces, none of the niche similarity and equivalency tests were significant.

### 3.2 Potential European distribution using source populations

**Figure 3:**
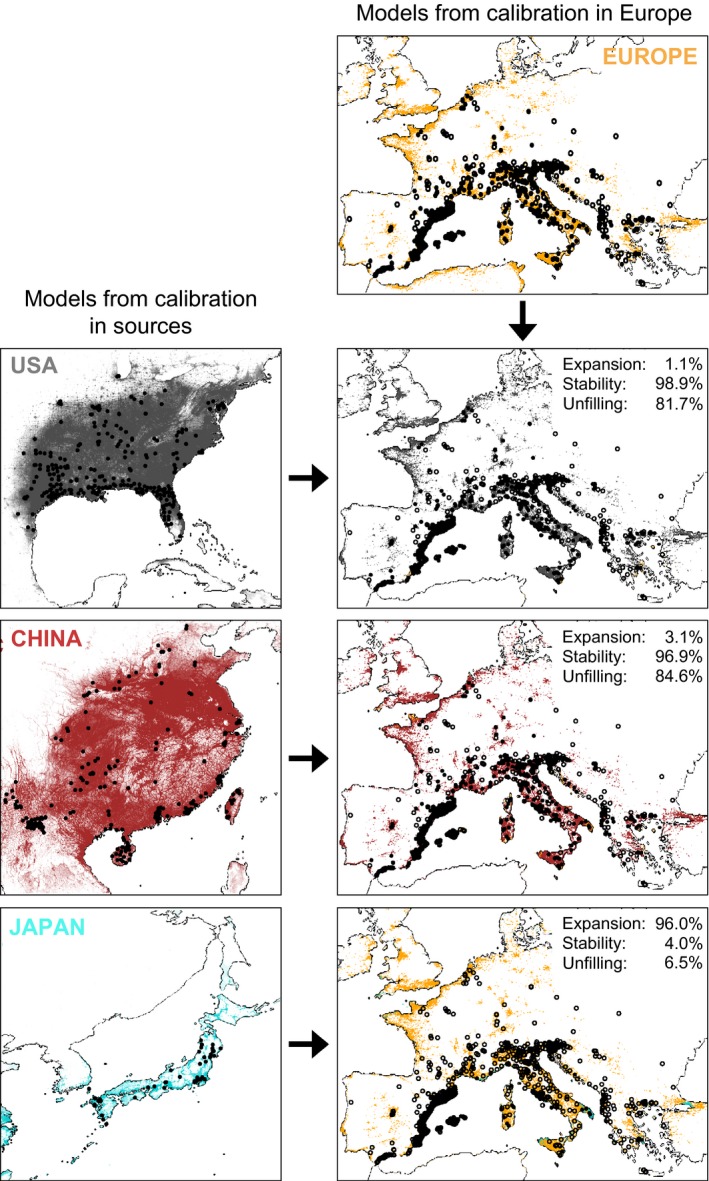
Niche‐based distribution modeling. The upper and left boxes, respectively, represent the results obtained from models calibrated in Europe (yellow) and in areas outside Europe: United States (gray), China (red), and Japan (blue). The right boxes indicate predicted areas of models calibrated in areas outside Europe projected into Europe (yellow: only predicted by Europe, gray: predicted by Europe and United States, red: predicted by Europe and China, blue: predicted by Europe and Japan). European occurrences are colored according to model predictions (white: not predicted, black: predicted). Niche changes scenarios: expansion, stability and unfilling represent agreement percentage between each pair of models

**Figure 4:**
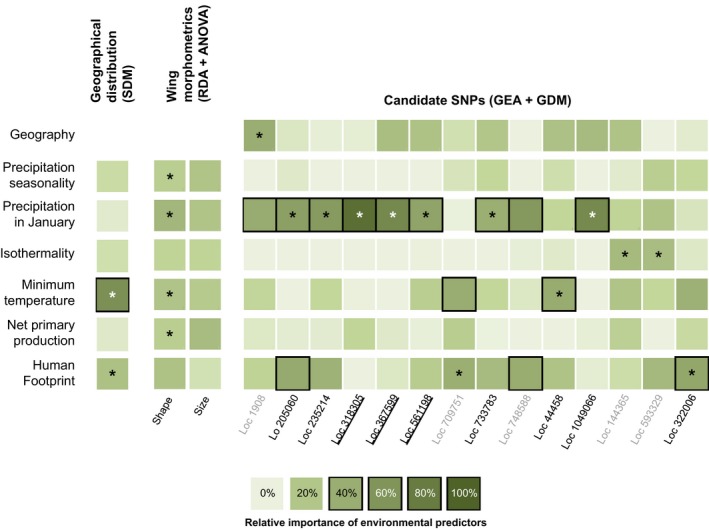
Relative importance of environmental predictors on geographical distribution, morphometric and adaptive genetic variation in Europe. The same six environmental predictors were used in niche‐based species distribution modeling (SDM; left), RDA‐based morphometric–environment correlations (center), and genotype–environment associations (GEA) and generalized dissimilarity modeling (GDM; right). Darker shading indicates greater relative importance, and relative importance ≥40% are surrounded by black boxes. Stars indicate the significance of tests. Morphometric traits: ANOVA results for one‐factor RDA models; SNP loci: % of GDM deviance for candidate SNP loci higher than % of GDM deviance for reference group; and environmental predictor inducing higher allelic turnover than reference group, geography and other environmental predictors (Table 1). Locus (Loc) name colored according to GDM results, gray: not candidate, black: candidate. Loci located in genes are underlined

The quality of distribution models was very high with an average TSS of 0.97, 0.90, 0.93, and 0.92 for Japan, China, United States, and Europe, respectively, and an average ROC of 0.99 for the four areas. Two environmental variables were the most relevant predictors of *Ae. albopictus* distribution: HF and MTP (Figure S5). The patterns of relative importance were different among regions, both being predictors for the geographical distribution in China and Europe, mostly HF for Japan and MTP for the United States. The model calibrated in Europe predicted 95% of total European occurrences (Figures 3 and 4). The potential European invasive range was also very well predicted by the models calibrated in China and United States (niche stability > 96%), but much lower than the total potential distribution predicted by sources (niche unfilling > 80%; Figure 3). Models fitted in Japan failed to predict the potential distribution in Europe with only 4% overlap in predicted areas (Figure 3).

### 3.3 Detection of adaptive SNP loci

The identification of adaptive SNP loci was carried out in three steps. We detected outlier loci correlated to environmental variables using two GEA methods (LFMM and RDADAPT) while controlling for genetic structure. We then used outlier loci detected by the two GEA methods in *F*_ST_‐based GDM. Loci showing a significant shift in allele frequencies along one relevant environmental predictor were considered as candidate loci. Those located in genes were considered as potentially involved in adaptation, and we predicted adaptive genetic composition in space using the relevant environmental predictor fitted.

Among the 6,506 SNPs analyzed, LFMM and RDA, respectively, discovered 279 and 471 significant associations with environmental variables. A total of 21 loci (133 SNPs) were detected by both methods (Table S3). Among 133 SNPs, 18 outlier SNPs were discovered by both GEA methods with a FDR of 5% (Table S3) that were distributed across 14 loci analyzed independently within GDM. All these loci had higher %GDM than the model performed with the reference group, supporting that population genetic structure was adequately accounted in GEA (Table 1). The partial allelic turnover of each locus in relation to each environmental variable and geographical distances revealed one locus mainly associated with geography (Locus 1908), thus representing a false positive (Table 1). The other 13 loci had an explicit allelic turnover for at least one environmental variable relative to the turnover for reference group and geography (in bold in Table 1, Figure S6). The environmental variable inducing the largest partial allelic turnover for each locus was considered as the most relevant predictor underlying changes in allele frequencies. The relevant predictor for most of the changes in genetic composition was PRJ (7 loci; Table 1, Figure S5). Allelic turnover was also observed in relation to temperature predictors (3 loci) and human footprint (3 loci). Among the 13 loci, 11 also show ≥ 40% of %GDM explained by one relevant environmental predictor (not for loci 144365 and 593329; underlined in Table 1). We retained nine candidate loci showing that the environmental predictor inducing the most significant allelic turnover also explained ≥ 40% of %GDM (not for loci 709751 and 748588; Table 1). Candidate loci are highlighted with both stars (largest allelic turnover) and boxes (≥40% of %GDM) in Figure 4.

**Table 1:** Signatures of environmental adaptation in the genome of *Aedes albopictus* European invasive populations

SNP information		Outlier SNPs (GEA)				GDM					Partial allelic turnover (GDM)						
Locus	Supercontig	LFMM (*N* = 551)	rdadapt (*N* = 551)	Common	SNP position	Npop (*N* ≥ 5)	Intercept	Null deviance	ΔDeviance	%GDM	Geography	PRS	PRJ	ISO	MTP	NPP	HF
1908	JXUM01S000008	15	1	1	499,740	49	0.18	230	39	17	0.75	0.03	0.59	0.00	0.14	0.01	0.15
44458	JXUM01S000095	2	2	2	539,477	50	0.15	185	11	6	**0.06**	**0.08**	**0.07**	0.06	**0.12**	0.00	**0.07**
					539,489												
144365	JXUM01S000410	14	2	2	304,164	47	0.18	158	16	10	**0.11**	0.00	**0.14**	**0.17**	**0.03**	0.02	**0.03**
					304,225												
205060	JXUM01S000625	1	1	1	30,435	54	0.16	199	38	19	**0.02**	0.08	**0.49**	0.00	0.00	0.01	**0.25**
235214	JXUM01S000771	1	1	1	61,551	59	0.19	250	41	17	0.00	0.00	**0.49**	0.00	**0.08**	0.00	**0.27**
318305	JXUM01S001117	9	1	1	23,481	52	0.22	201	22	11	**0.01**	0.00	**0.64**	0.05	0.05	**0.08**	0.00
322006	JXUM01S001159	3	3	2	165,202	50	0.20	196	11	5	0.03	0.00	0.02	0.06	**0.12**	0.00	**0.15**
					165,212												
367599	JXUM01S001412	5	3	1	223,015	49	0.11	225	72	32	**0.28**	0.03	**1.00**	0.00	0.00	**0.07**	**0.03**
561198	JXUM01S002618	1	1	1	23,932	58	0.14	316	31	10	**0.19**	0.00	**0.23**	0.07	**0.09**	0.02	**0.19**
593329	JXUM01S002886	10	2	2	184,498	48	0.17	125	15	12	0.00	**0.10**	**0.14**	**0.15**	**0.00**	0.00	**0.10**
					184,529												
709751	JXUM01S004010	1	1	1	142,549	54	0.20	177	21	12	**0.08**	0.05	0.01	0.00	**0.13**	**0.04**	**0.15**
733783	JXUM01S004273	1	2	1	12,419	57	0.11	365	83	23	**0.14**	**0.18**	**0.57**	0.00	**0.10**	0.00	**0.22**
748588	JXUM01S004403	7	1	1	112,864	53	0.00	152	25	16	**0.23**	0.09	**0.20**	0.02	0.03	0.00	**0.25**
1040966	JXUM01S011360	4	2	2	17,728	51	0.17	187	26	14	**0.17**	0.00	**0.47**	0.02	0.00	**0.06**	**0.07**
					17,734												
Reference group							0.36	236	3	1	0.00	0.05	0.02	0.09	0.01	0.02	0.00

Note

Outlier loci detected by GEA (14 loci, *Q*‐value < 0.05), with the position of the 18 common SNPs (=outlier SNPs). *Q*‐values for all SNPs detect by GEA (*N*_loci_ = 21, *N*_SNPs_ = 133) are in Table S3. Results of GDM for the 14 loci, with partial allelic turnover for each locus in relation to each environmental variable and geographic distance. GDM significance: SNP loci with % deviance > % deviance for reference group and allelic turnover in relation to each environmental predictor > allelic turnover for reference group and geography are indicated in bold. Underlined: environmental predictors explaining ≥40% of GDM deviance.

**Figure 5:**
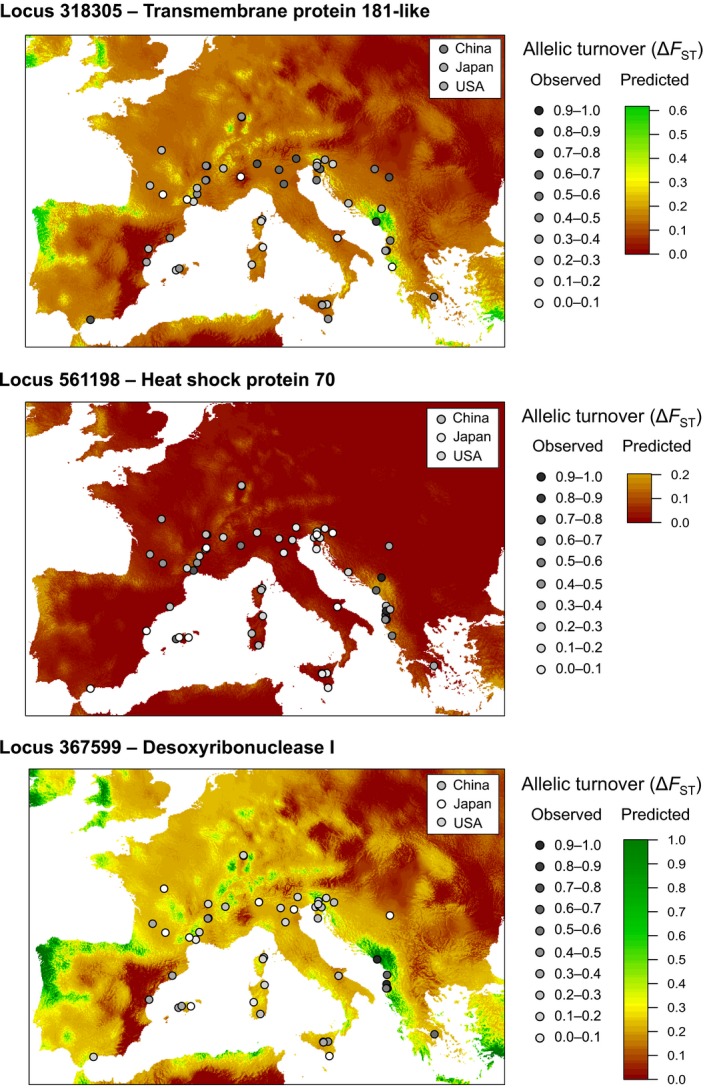
Predicted spatial variation in population‐level adaptive genetic composition from GDM. Map font colors represent gradients in allelic turnover derived from transformed environmental predictors (precipitation in January). Dot colors represent gradient in allelic frequencies observed in European invasive populations, with insert indicating allelic frequencies in source populations. For each locus, the partial allelic turnover along the precipitation gradient in January is the predicted Δ*F*_ST_. Partial allelic turnovers for the 14 candidate SNP loci in relation to geographic distances and each environmental variable are presented in Figure S6

Among the nine candidate loci, three were located in genes. Locus 318305 is located in AALF001258 encoding a transmembrane 181‐like protein involved in toxic substance binding, Locus 367599 is located in AALF004989 orthologous to *DNase I*, and Locus 561198 is located in AALF012056 that has more than 96% sequence homology with mosquito heat‐shock 70‐kDa protein cognate 1 (*Hsc70‐1*) belonging to the *Hsp70* family. The expected variation in genetic composition for these three adaptive loci was predicted using GDM results and PRJ raster data at the European scale (Figure 5). The expected pattern was similar among adaptive loci, with higher frequencies of the alternative allele in areas with high precipitation during winter but differed in the magnitude of allelic turnover. Despite large %GDM explained by PRJ in fitted models, the predicted dissimilarities did not well fit the observed dissimilarities (Figure S7). The two alleles were already present before introduction in Europe, but most European populations show different allele frequencies from their source (Figure 5).

### 3.4 Wing morphometric variation

**Figure 6:**
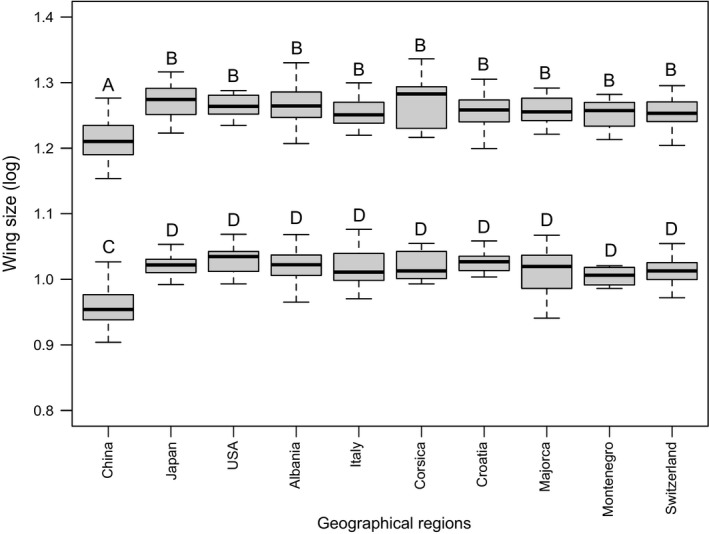
Wing size variation among *Aedes albopictus* populations. Each males–females comparison is significant. Letters represent the results of ANOVA for comparisons among main geographical regions, with different uppercase letters indicating significant tests. Sample sizes (male/female): United States = 16/11; China = 50/55; Japan = 27/21; Italy = 18/63; Albania = 55/53; Corsica = 8/14; Majorca = 11/7; Croatia = 19/20; Montenegro = 9/11; Switzerland = 13/7. ANOVA results for comparisons among populations are in Figure S8B

Wing shape differentiation between males and females accounted for 33% of total wing shape variation (PC1; Figure S8A). Although source populations (China, United States) can be differentiated based on wing shape (PC2, 16% of total wing shape variance), this analysis revealed low variation among European invasive populations. Even if low, wing shape variation among geographical regions and populations was significant for females (MANOVA, respectively, *df* = 9, *F* = 2.32, *p* < 2.e−16; *df* = 18, *F* = 1.71, *p* < 2.e−16) and males (MANOVA, respectively, *df* = 9, *F* = 2.08, *p* < 2.e−16; *df* = 18, *F* = 1.80, *p* < 2.e−16). Similarly for females and males, all the populations located in temperate conditions (United States, Japan, Europe) were different from those in subtropical conditions (China; ANOVA, females: *df* = 9, *F* = 16.26, *p* < 2.e−16; males: *df* = 9, *F* = 11.94, *p* < 2.e−16). Wing size variation among populations was also significant (ANOVA, females: *df* = 18, *F* = 25.27, *p* < 2.e−16; males: *df* = 18, *F* = 14.03, *p* < 2.e−16; Figure 6 and Figure S8B). As morphometric variation among European populations was low, we also found low correlation between morphometric traits and environmental variables. RDA on wing shape revealed that 6.9% of total variation was constrained by environmental conditions (ANOVA, *F* = 4.22, *p* = .001). One‐factor RDA models indicated that all the environmental variables constrain equivalent proportion of wing shape variation (ranging from 0.7% to 2.7%, Figure 4). RDA on wing size revealed only 1% of variation constrained by environmental conditions and was not significant (ANOVA, *F* = 0.60, *p* = .748).

## 4 DISCUSSION

### 4.1 Niche conservatism in *Aedes albopictus*

Niche conservatism during the invasion process of *Ae. albopictus* has been examined previously (Cunze et al., 2018; Hill et al., 2017; Medley, 2010). These studies suggested that invasive populations' niche ranges (Europe, North America, South America, Africa) differ from those of native Asian populations (Cunze et al., 2018; Medley, 2010). However, niche comparisons did not account for the origin of introduced populations while this knowledge is being increasingly available (Kotsakiozi et al., 2017; Sherpa, Blum, Capblancq, et al., 2019). Here, we compared the environmental niches of European introduced populations with the one of their US (from ancestral Japan origin that was included in the analysis) and Chinese sources (Sherpa, Blum, Capblancq, et al., 2019). The comparison of niches in reduced environmental space revealed that the two source populations for the European invasion have broad, different but overlapping environmental niches (Figure 2a), as previously reported (Cunze et al., 2018; Medley, 2010). The distribution of European populations in this space reveals niche differences between primarily introduced populations and their source populations, with centroids shift related to winter climate for Albania and human pressure for North Italy (Figure 2a).

Regarding subsequent introductions, the colonization of particular environmental space in Europe seems to be related to specific subgroups of source populations. Indeed, secondary introductions can occupy niches different from those of primarily introduced populations they originate from (Albania, North Italy, and Central Italy) but that still overlap the vast niche of ancestral source populations (the United States and China), which could support niche conservatism. Nonetheless, we found the European invasive niche neither more equivalent nor more similar to one of the source niche than random expectations, supporting neither conservatism nor shift between invaded and source niches. Because models calibrated in source populations are expected to accurately predict the current European invasive range if niche is conserved (Wiens & Graham, 2005), we evaluated the different possible scenarios of niche shift that can explain niche differences using SDM results. Both niche expansion and niche unfilling explain why the niches of invasive populations do not overlap with the niche of their sources. While niche expansion predicts a species to occupy different environmental areas (niche shift), niche unfilling indicates that invasive populations do not occupy all areas predicted by the sources, regardless of niche stability. Niche stability is the proportion of overlapping niches, which indicates the tendency of populations to retain their niche (niche conservatism; Guisan et al., 2014). We found a high proportion of niche stability (>96%) and niche unfilling (80%–85%), supporting niche conservatism between European invasive populations and their sources (China, United States), but also indicating that *Ae. albopictus* does not (yet) occupy all the suitable areas available in Europe, which could be related to the short time since introduction in Europe (40 years). This result is congruent with those found by Cunze et al. (2018) and suggests that niche conservatism is the typical pattern in *Ae. albopictus* invasion process.

### 4.2 Environmental adaptation after introduction in Europe

Niche comparisons, supporting niche conservatism, do not suggest that the invasive range expansion within Europe required new evolutionary adaptations. Nonetheless, examining the variation at wing morphometric traits and genomic loci, we found evidence for signatures of selection after the introduction of *Ae. albopictus* in Europe. Wing size and shape weakly differ among European invasive populations (Figure 6, Figure S8) and are not correlated with environmental variables (1%–6%). Furthermore, we found that the wing size of European invasive populations (36.6°N–48.8°N) do not differ from those of Japan and United States (35.9°N and 33.7°N, respectively). Our results are consistent with studies showing among‐population variation but no differences across latitudes of temperate areas in *Ae. albopictus* (Armbruster & Conn, 2006; O'Donnell & Armbruster, 2009; Urbanski et al., 2012). Wing traits measurements were performed under common garden conditions, thus reflecting only genetic differences. The absence of variation in wing size and shape could reveal that these traits are neutral. However, a previous analysis in *Ae. albopictus* revealed wing size clines among tropical, subtropical, and temperate native ranges (Sherpa, Blum, & Després, 2019). Furthermore, all temperate invasive and native populations differ from one of the European source located in more southern latitudes (China: 23.1°N), suggesting postintroduction changes in the size of individuals as Albanian populations were introduced from China (Sherpa, Blum, Capblancq, et al., 2019). Our results in *Ae. albopictus* are thus consistent with parallel climate‐mediated selection on insect wing size in the native and invaded ranges located in similar temperate latitudes (Blanckenhorn & Demont, 2004). The climatic variation encountered during the range expansion across ~12° of latitude within Europe is probably not strong enough to induce variation in this trait.

Searching for signatures of selection within the genome of *Ae. albopictus*, we reveal three adaptive loci associated with precipitation during cold periods (Figures 4 and 5). Cold and drought are crucial factors influencing the survival of overwintering insects (Block, 1996). *Aedes albopictus* winter survivorship in cold environments is determined by the photoperiodic induced diapause of eggs (Hanson & Craig, 1994; Hawley, 1988), which has a complex molecular basis (Armbruster, 2016). In Europe, *Ae. albopictus* overwinters for 6 months from autumn to the next spring and can experience minimum winter temperature ranging −13.1–11.8°C and precipitation during the coldest month ranging from 18 to 227 mm (from occurrence distribution; Table S2). Physiological experiments evaluating the relationships between water availability and cold hardiness have shown higher survival chance in *Ae. albopictus* adults (Zhang et al., 2019) or other insect eggs (Qi, Wang, Xu, & Kang, 2007) when exposed at subzero temperatures for a short period as long as they were exposed with water. However, long‐time exposure of insect eggs at low temperature in dry soil may be an essential factor of egg mortality due to their frost susceptibility (Qi et al., 2007). Cold and dry environments thus represent potential intense selective pressure on *Ae. albopictus* egg overwintering survival. Accordingly, we detected adaptive genetic variation in relation to precipitation during cold periods in European populations, suggesting postintroduction adaptive changes to overcome these unsuitable environmental conditions. For example, the winters experienced in Albania and Montenegro are cold and wet (Petrić et al., 2018), with an average of 124 mm of precipitation in January compared to an average of 52 mm for other locations in Europe for the same range of minimum temperatures during the coldest month, and populations in these regions show the highest proportion of mutations in genes detected as putatively under divergent selection (Figure 5). One of the adaptive loci is located in a gene encoding a heat‐shock protein (Hsp) homologue. Hsps are often upregulated during insect diapause (Chen, Kayukawa, Monteiro, & Ishikawa, 2005; Gkouvitsas, Kontogiannatos, & Kourti, 2009; Rinehart et al., 2007; Rinehart et al., 2007; Yocum, 2001; Yocum et al., 2005). The homologous protein detected is the Hsp70 cognate, *Hsc70*, which is upregulated in a wide range of diapausing insects (Chen et al., 2005; Gkouvitsas et al., 2009; Rinehart et al., 2007; Rinehart et al., 2007; Yocum, 2001; Yocum et al., 2005) and during acute cold exposure in *Ae. albopictus* (Zhang et al., 2019). In addition to the different adaptive strategies to prevent damages induced by ice formation (Armbruster, 2016; Kreß, Kuch, Oehlmann, & Müller, 2016), Hsps could contribute significantly to the invasive success of *Ae. albopictus* by increasing overwintering survival of eggs at low temperature.

Examining the spatial distribution of genetic variation at candidate loci, *Ae. albopictus* populations established in Albania did not expand a lot (Montenegro, Serbia, and Greece; Sherpa, Blum, Capblancq, et al., 2019). Extrapolating the predicted allelic frequency from precipitation during winters, this result suggests that not only the political and commercial isolation, and the prolonged history of reduced genetic diversity, have restricted Albanian populations to their initial area of introduction (Sherpa, Blum, Capblancq, et al., 2019) but also the low proportion of suitable areas for those populations in Europe. Bottlenecked populations generally have low genetic diversity, which should reduce their fitness and adaptive potential (Lee, 2002; Prentis, Wilson, Dormontt, Richardson, & Lowe, 2008; Rius & Darling, 2014). However, several mechanisms related to the demographic history of European invasive populations could have promoted adaptation. An alternative explanation to selection for the observed genetic shift between Albania and China is a purely demographic effect during the invasion process. The substantial bottleneck during the introduction in Albania (Sherpa, Blum, Capblancq, et al., 2019) could have allowed the expression of beneficial alleles, previously masked by the expression of other alleles lost during founder event (Blows & Hoffmann, 2005). Primarily introduced populations in North Italy did not widely expand (Slovenia, Switzerland) while those established in Central Italy, which received genetic input from China and North Italy dispersed throughout the western Mediterranean basin (Sherpa, Blum, Capblancq, et al., 2019). Founding admixture generated novel genetic combinations allowing populations to establish in various niches in the environmental space of their two sources (Figure 2), further supporting the role of multiple introductions in promoting invasiveness (Dlugosch, Anderson, Braasch, Cang, & Gillette, 2015; Rius & Darling, 2014).

## 5 CONCLUSIONS

Following our initial reports on the role of genetic diversity in invasive populations (propagule pressure, genetic admixture) and preexisting adaptations within the native range (cold adaptation; Sherpa, Blum, Capblancq, et al., 2019; Sherpa, Blum, & Després, 2019), we evaluated whether niche characteristics could also be an essential predictor of *Ae. albopictus* invasive success in Europe. We confirm that niche conservatism is the typical pattern in *Ae. albopictus* invasion process (Cunze et al., 2018), which seems to be dominant among invasions in similar climate areas (Guisan et al., 2014). This result together with the apparent low conservatism between the environmental niches in Europe and Japan suggests that invasive populations retain the niche of their US source where the niche shift occurred, as *Ae. albopictus* has not directly been introduced in Europe from Japan (Sherpa, Blum, Capblancq, et al., 2019). However, niche differences observed between US populations and their sources (Figure S4), or the native Asian range (Medley, 2010), are probably due to niche unfilling (Cunze et al., 2018). Despite niche conservatism characterizes the European and North American invasions, the rapid evolution of traits (Europe: present study; Kreß et al., 2016, United States: Urbanski et al., 2012; Armbruster, 2016; Medley, Westby, & Jenkins, 2019) suggests genetic shift from standing variation in response to new selective pressures encountered in the invaded area. Adaptive shifts could relate to niche differences induced by niche conservatism. Indeed, preexisting adaptation in source populations can promote the colonization of a wide range of habitats under the same climate, such as the cold adaptation for invading temperate regions observed in *Ae. albopictus* (Hawley, 1988; Sherpa, Blum, & Després, 2019). The allelic shifts from standing variation observed after introduction reflect fine‐tuning adaptations to the local conditions encountered in the introduced range, already present in their ancestral niche.

The adaptive potential of invasive populations is likely to represent an essential component of the invasion process. In the present study, we show that the geographical distribution of invasive populations correlates with their adaptive genetic composition. The absence of *Ae. albopictus* in cold and drought areas could either suggest that the short time frame since introduction was not sufficient for *Ae. albopictus* to reach these areas or that adaptation required to invade these regions did not or will not occur. These two hypotheses have contrasted implications for studies predicting the potential distribution of invasive species. Nonetheless, invasive species distribution models are classically predicted from occurrence and environmental data only and do not account for their adaptive and thus invasive potential. Observed genetic shift and niche conservatism together with the estimation that about 80% of *Ae. albopictus* potential geographical distribution is yet unfilled, suggest further spread of *Ae. albopictus* in Europe. The expected allelic turnover at adaptive loci at the scale of the fitted gradient could be used to refine the degree of suitability for the establishment of invasive populations, as well as in species distribution under climate change (Peterson, Doak, & Morris, 2019).

## CONFLICT OF INTEREST

None declared.

## AUTHOR CONTRIBUTION

SS designed the study. SS, LD, MA, BA, CA, HB‐C, RB, MB, X‐GC, RE, EF, CF, IHI, KK, SK, FM, IP, DP, RT, NT, GMV‐P, EV, GV, and XZ designed the sampling and collected the samples. SS performed DNA laboratory work and morphometrics data acquisition. JR collected environmental data. SS and TG performed mosquito rearing, with support from LD and FL. SS analyzed genomics, morphometrics, and environmental niche data. MG performed species distribution models. SS wrote the manuscript, with input from MG, JR, LD, RE, and DP.

## Data Availability

ddRADseq sequences are available at the European Nucleotide Archive (http://www.ebi.ac.uk/ena) through accession numbers PRJEB20192 and PRJEB31109.
