# Supplementary material for: Predicting the success of an invader: Niche shift versus niche conservatism
Source: Ecol Evol. 2019 Oct 25;9(22):12658–75. doi: 10.1002/ece3.5734 (PMC6875661; doi:10.1002/ece3.5734)
Supplement: Supplementary file 1 [file ECE3-9-12658-s001.pdf]

SUPPLEMENTARY FIGURES

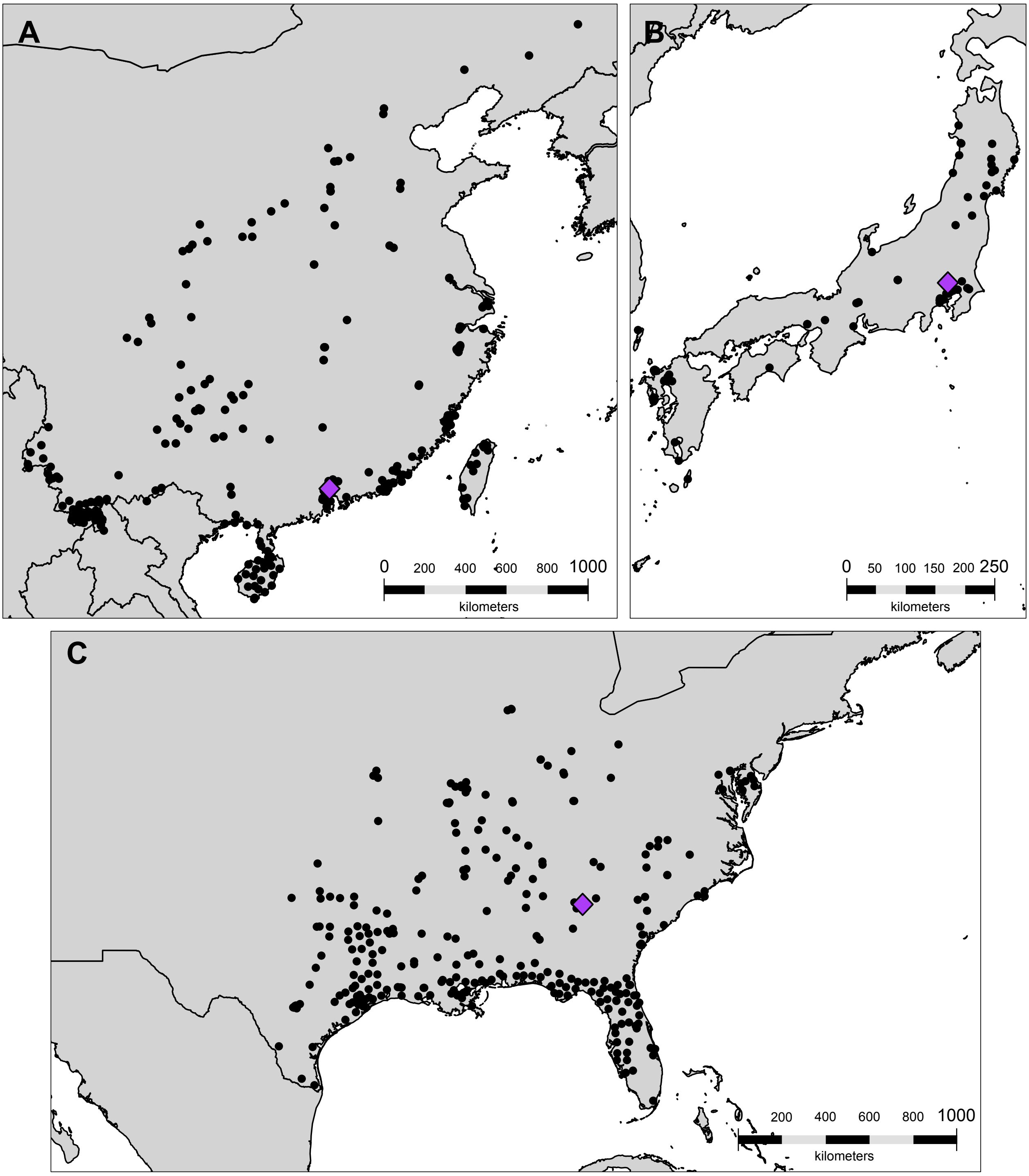

Figure S1 Occurrence data of *Aedes albopictus* source populations. Geo-referenced occurrences used for niche comparisons in China: N = 265, Japan: N = 81, and USA: N = 275 (occurrences before 1990 = date of introduction in Europe from USA). Black: observation data, violet: locality with available ddRADseq genomic data (Sherpa et al. 2019a).

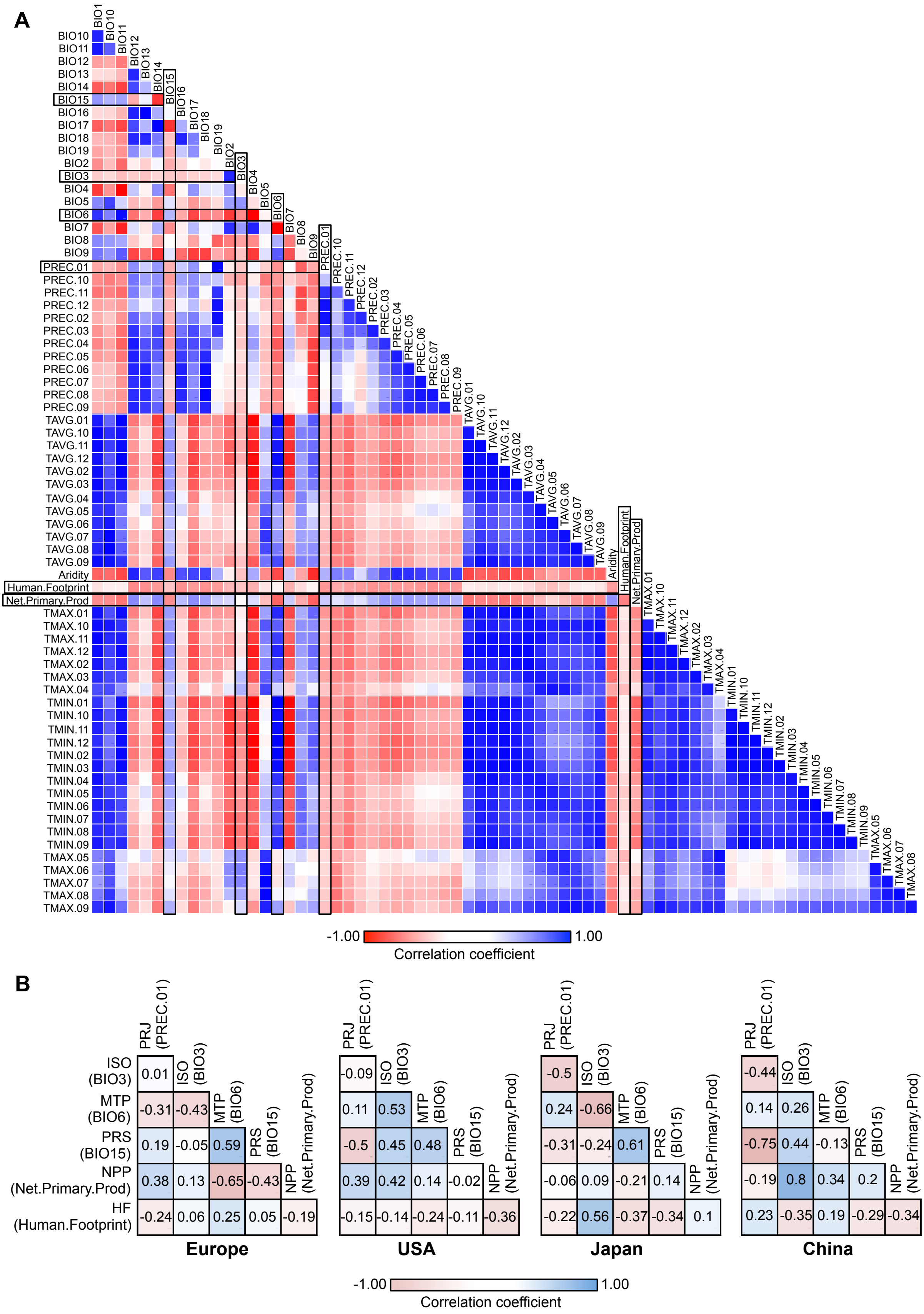

Figure S2 Environmental variables selection. A) Pearson's correlation between the 70 environmental variables considered using all occurrences (Europe, China, USA, Japan). Black boxes surround uncorrelated selection variables. **B**) Pearson's correlation between the 6 selected environmental variables for each geographical region: precipitation in January (PRJ, PREC01), isothermality (ISO, BIO3), mean temperature of the coldest month (MTP, BIO6), precipitation seasonality (PRS, BIO15), net primary production (NPP), human footprint (HF). Selected variable present correlation coefficient < 0.50 among geographical regions. Font colors according to coefficients of correlation, red: $R = -1.00$, blue: $R = 1.00$.

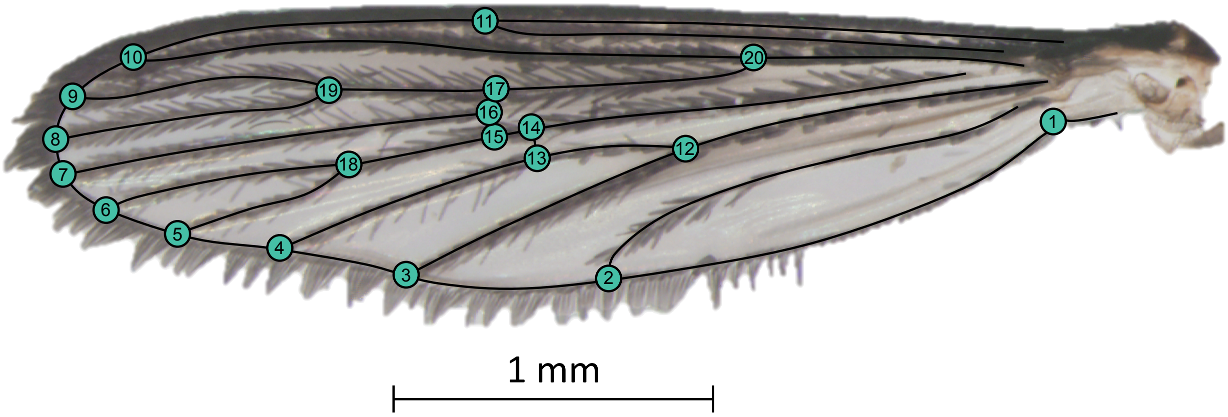

Figure S3 Wing geometric morphometrics. Localization of landmarks used to represent wing variation (from Sherpa et al. 2019b). Median vein (LM1); anal vein (LM2), distal end of cubital vein 2 (LM3) and vein 1 (LM4), distal end of media 3 and media 4 (LM5), distal end of media 1 and media 2 (LM6), distal end of radial branches 4 and 5 (LM7), radial branch 3 (LM8), radial branch 2 (LM9), distal end of the radius (LM10) and intersection of costa (LM11). Origin of cubital 1 (LM12), midpoint branch of cubital 3 (LM13), medio-cubital cross vein (LM14), midpoint branch of medial vein (LM15), radio-medial cross vein (LM16), midpoint branch of radial vein (LM17), radio-sectoral vein (LM18), origin of radius branches 2 and 3 (LM19), radial sector (LM20).

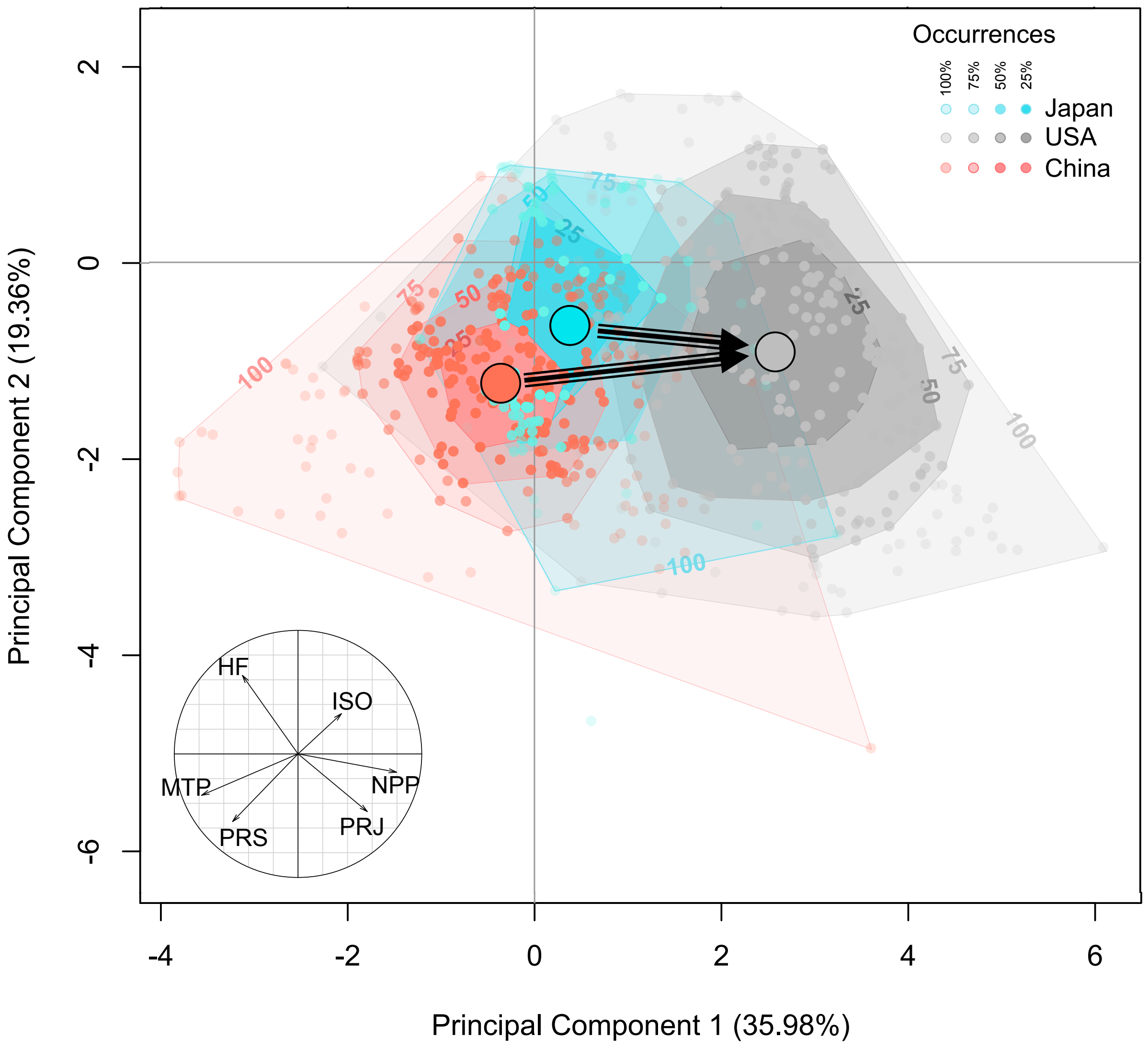

Figure S4 Environmental space of source populations. The convex hulls indicate the prevalence (25, 50, 75 and 100% of sites included) of the environmental conditions. Occurrences are indicated with small dots and centroids with big dots. Black arrows linking centroids represent the origins of USA populations. The correlation circle indicates the importance of environmental variables on the two first axes of the PCA (55% of the total variance): PRJ (precipitation in January), PRS (precipitation seasonality), MTP (minimum temperature of the coldest month), ISO (isothermality), NPP (net primary production), and HF (human footprint).

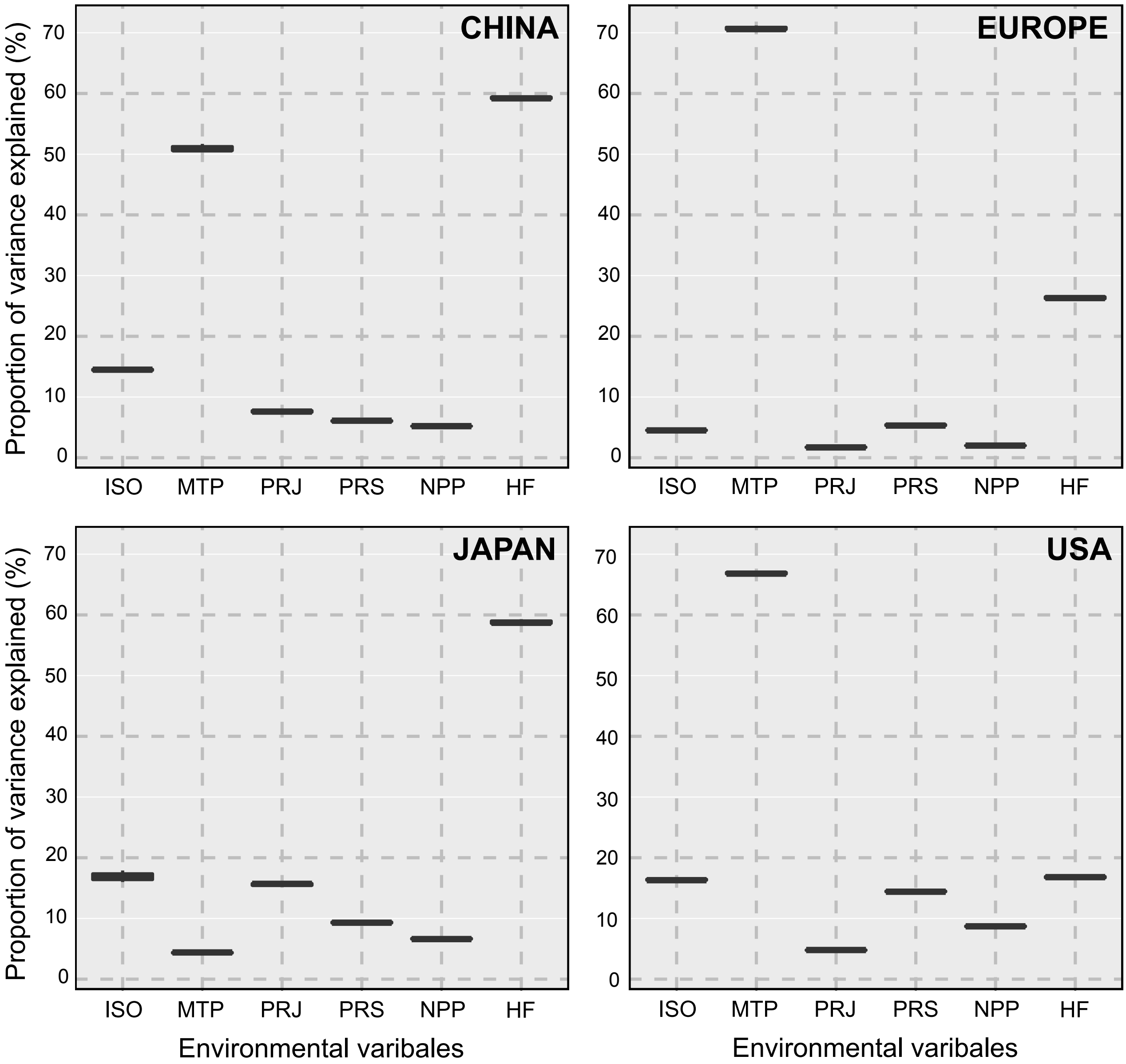

Figure S5 Environmental determinants of *Aedes albopictus* geographical distribution. Relative importance of the six environmental variables used to project the potential distribution of *A. abopictus* in Europe (Figure 3) for models obtained from calibration in China, Europe, Japan, and USA.

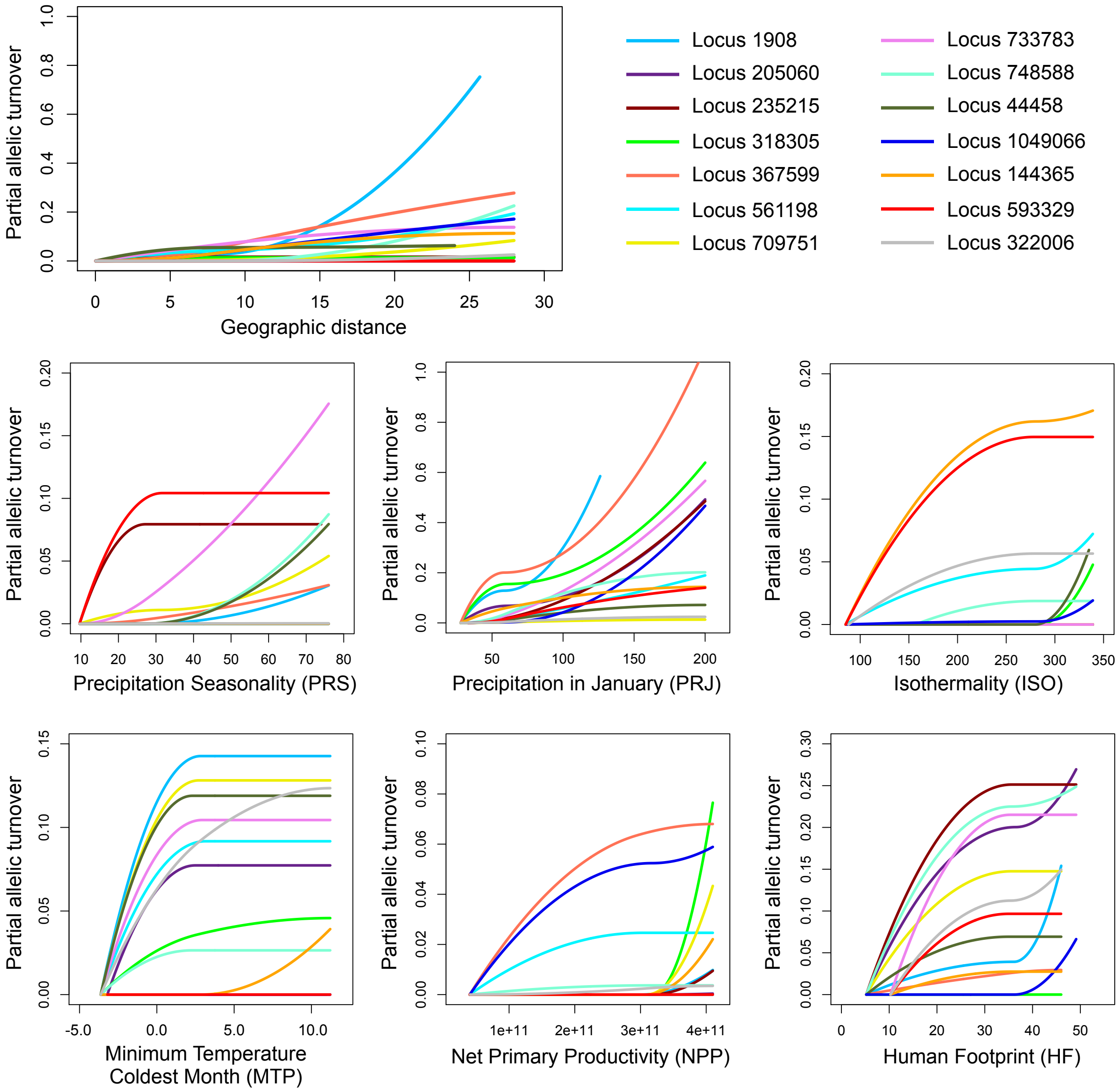

Figure S6 Allelic turnover of outlier loci. Allelic turnover functions for the 14 SNP loci detected as outliers by GEA (LFFM and RDA) in relation to each environmental variable and geographic distance. For each locus, the fraction of generalized dissimilarity modeling (GDM) deviance implied by each environmental variable is given in Figure 4, and the magnitude of allelic turnover in Table 1.

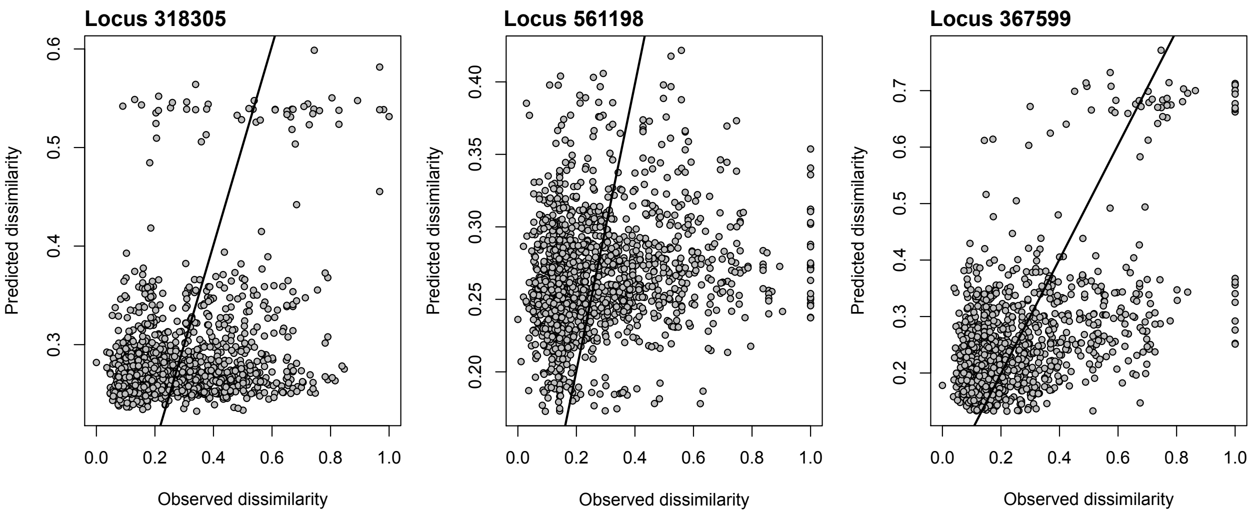

Figure S7 Predicted allelic distances between sites for adaptive genetic variants. Adaptive genetic variants i) were detected as outliers by GEA (overlapping between LFFM and RDA, and with a *Q*-value < 0.05; Table 1, Table S3), ii) show higher percentage of deviance explained by environment than for the reference group of SNPs (200 random SNPs as representative of the neutral genetic structure; Table 1), iii) show variation constrained mainly by one environmental variable (>40% of GDM deviance; Figure 4), iv) show higher allelic turnover along this environmental gradient than the reference group and than along geographic distance (Table 1, Figure S5), v) are located in protein coding region. Predicted changes in allele frequencies were used to project the putative distribution of adaptive genetic variation in Europe (Figure 5).

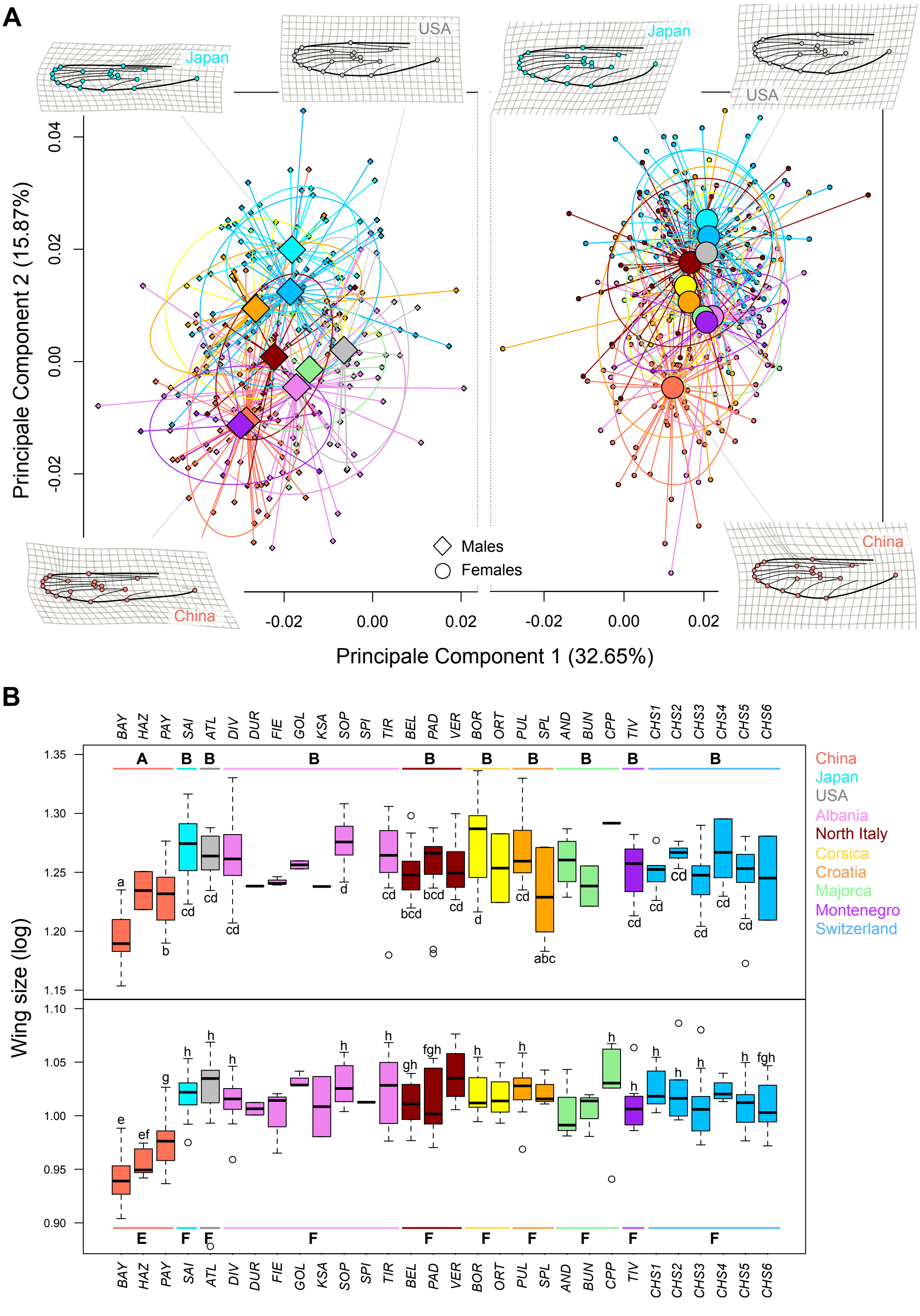

Figure S8 Wing mophometric variation among *Aedes albopictus* populations. A) Principal Component Analysis (PCA) on Procrustes coordinates, with 49% of total variance accounted by the first two PCs. Schematic representation of wing landmark deformations on the same axes reconstructed from grids and vectors are shown for source populations (China, USA, Japan). Solid lines have been added for better visualization. **B**) Wing size variation among populations for males and females (females: top, males: bottom). Each population males-females comparison is significant. Letters represent the results of ANOVA with different letters indicating significant tests (uppercase: comparisons among countries, lowercase: comparisons among populations with N ≥ 5). For comparisons among regions, male/female sample sizes: USA=16/11; China=50/55; Japan=27/21; Italy=18/63; Albania=55/53; Corsica=8/14; Majorca=11/7; Croatia=19/20; Montenegro=9/11; Switzerland=13/7. For comparisons among populations, sample sizes are given in Supplementary Table S1.
